# Accessory nipple reconstruction following a central quadrantectomy: a case report

**DOI:** 10.1186/1757-1626-2-32

**Published:** 2009-01-08

**Authors:** Stefano Magno, Daniela Terribile, Gianluca Franceschini, Cristina Fabbri, Federica Chiesa, Alba Di Leone, Riccardo Masetti

**Affiliations:** 1 Department of Surgery, Breast Unit, Catholic University Policlinico "A. Gemelli", Largo Agostino Gemelli, 00168 Rome, Italy

## Abstract

**Introduction:**

nipple dichotomy (or intra-areolar polythelia) is a rare congenital malformation in which one or more supernumerary nipples are located within the same areola.

A case of a woman undergoing a central quadrantectomy with a contralateral supernumerary nipple used for reconstruction is reported. No other report in the Literature, according to our search, has focused on reconstructive use of an accessory nipple after breast conserving surgery.

**Case presentation:**

the patient is a 73 year-old Caucasian woman, who two years earlier underwent a lower-outer left Quadrantectomy plus axillary sampling and radiation therapy for a 2,2 cm lobular carcinoma with no lymph node involvement.

A routine follow-up assessment showed an important fibrotic change on the operated breast, just across the infra-mammary fold; at a breast Magnetic Resonance Imaging, a 1,5 cm area in retroareolar position, suspicious for local recurrence, was evident.

An open biopsy was therefore performed, under local anaesthesia, including the nipple-areolar complex to realize a central Quadrantectomy with a Grisotti procedure; a congenital dichotomic nipple in the contralateral breast was then used to repair the defect through a "nipple-sharing" technique. The final histological examination reported a fibrotic mastopathy without atypias.

**Conclusion:**

in this case, the "nipple-sharing" technique has allowed in the same time the correction of a rare congenital defect and provided the surgeon with a supernumerary nipple to be used in the immediate reconstruction after breast conserving surgery.

## Introduction

Breast cancer located in the retroareolar part of the breast are still treated in many centers with radical mastectomy; the introduction in recent years of oncoplastic techniques has allowed the preservation of the affected breast with ample removal of the neoplasm along with the nipple-areolar complex, whenever needed, yielding as safe oncological results as more invasive procedures.

We report a case of a patient, in whom the presence of a suspicious local recurrence after a conservative approach for lobular carcinoma was associated with an unusual accessory nipple in the contralateral breast, which has allowed the immediate reconstruction of the surgical defect through a new areola obtained from a skin island of the ipsilateral breast together with a new nipple from the right side.

## Case presentation

**Figure 1:**
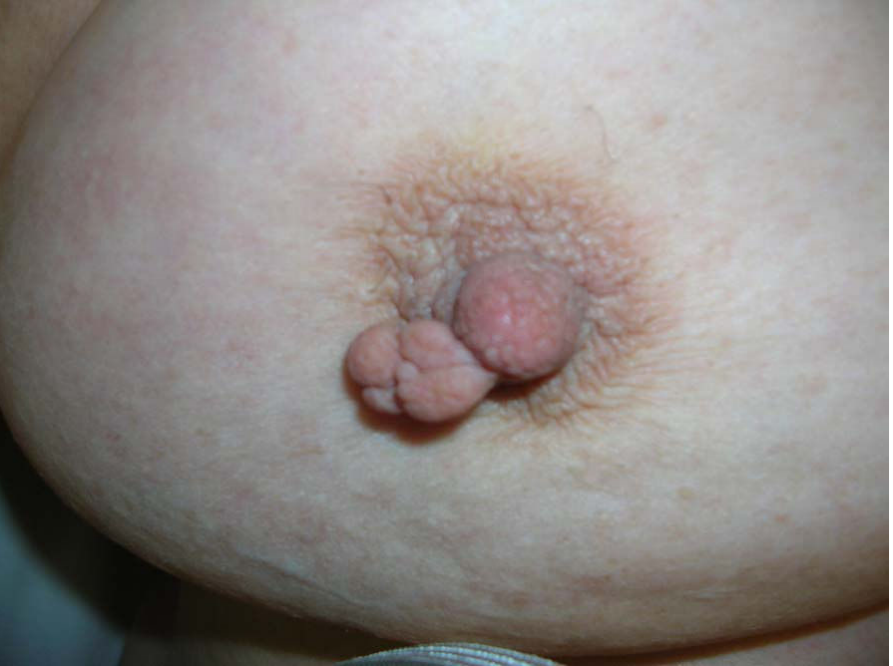
**Congenital abnormality known as nipple dichotomy or intra-areolar polythelia (description in the text)**.

The patient, a 73 year-old Caucasian woman, was born with a supernumerary nipple within a unique areola in her right breast, which was never reported to the physician, though she occasionally complained with a burning tenderness in that area, due to the contact with the bra (Fig. 1). This condition is known as "intra-areolar polythelia".

When she was 71, the patient noted an asymptomatic lump of the left breast. A mammogram revealed a lesion with irregular margins in the inferior part of the gland, 2 cm in size.

An ultrasound confirmed that finding and did not show any other suspicious finding either in the axilla and in the right breast.

No history of breast cancer in her first degree relatives, personal history of breast pathology or other remarkable medical or surgical facts were reported by the patient. Her menarche was at age 18; menses stopped spontaneously at age 52. No hormonal treatment was conducted in the past.

At physical examination, a 2 cm lump across the inferior quadrants of the left breast, not fixed to the chest wall but slightly retracting the overlying skin was found. No regional adenopathy, both in the axillary and supraclavicular basins, was detected.

The staging exams (chest X-ray, bone scintigraphy, liver ultrasound) did not show any distant metastases.

She underwent a lower-outer left Quadrantectomy, including a broad skin area macroscopically infiltrated and retracted, plus axillary sampling, in local anaesthesia, using an elliptical "comma" incision. By remodelling the gland, a normal shape of the operated breast was restored.

Deep dermis resulted microscopically involved by carcinoma, while the margins of resection, deep pectoralis fascia included, showed no tumour cells within 5 mm from the inked surface. Based on the histological results (lobular carcinoma pT2 N0), the patient underwent Radiation therapy and began an hormonal therapy with aromatase inhibitors (anastrazole).

Eighteen months later, while she was still on her hormonal therapy, a routine follow-up assessment with ultrasound and mammogram (October 2006) showed an important fibrotic change on the operated breast, particularly in the irradiated field, just across the infra-mammary fold.

A breast Magnetic Resonance Imaging was then performed, which revealed a left sided 1,5 cm area in retroareolar position, suspicious for local recurrence, along with an epidermal swelling of the entire breast.

At clinical examination, no significant findings, beside the already noted right accessory nipple and a significant left nipple retraction, were registered.

A ultrasound-guided breast fine needle biopsy failed to rule out malignant cells in the suspicious area; a Positron Emission Tomography (PET) similarly did not show any pathological finding, even in the left breast.

An open biopsy was anyway suggested by the radiologist, which, previous patient's informed consent, included the nipple-areolar complex (NAC) to realize a central Quadrantectomy using a Grisotti procedure, under local anaesthesia. This technique is often used in our Center, since it is quite simple and offers excellent cosmetic results; with the patient in the sitting position, a circle is drawn along the borders of the areola. Another circle is drawn below the areola and lines from the medial and lateral sides of the two circles are connected laterally on the inframammary fold.

**Figure 2:**
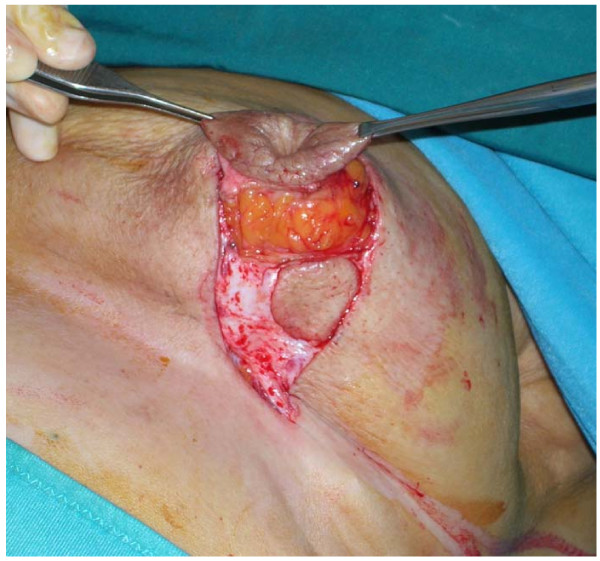
**Excision of the nipple-areola complex along with underlying parenchyma (central quadrantectomy); the lower skin circle will replace the excised areola**.

Incisions are made along the drawings and the skin below the areola is excised, with the exception of the one included in the lower circle. The NAC with the underlying cylinder of parenchyma is completely excised, while the skin-glandular flap mobilized from the inferior lateral pole of the residual gland is used to create the new areola (Fig. 2). The flap is incised medially down to the pectoralis fascia and separated from the latter to allow for adequate rotation and advancement. It is then sutured to the gland stump superiorly, and the circular area of preserved skin is sutured to replace the excised areola.

Particularly in this case, provided the post-radiation changes of the operated breast, care should be taken to avoid excessive devascularization of the skin-glandular flap, to minimize the risk of ischemic injury to the neo-areola.

At this point, a nipple-sharing technique has been used in the immediate reconstruction of the nipple: a small disc of epidermis has been excised from the centre of the neo-areola on the left breast and a graft of the supernumerary nipple of the right one has been sutured to the de-epithelialized area with a few absorbable stitches. The donor site defect has been sutured with the same absorbable material.

**Figure 3:**
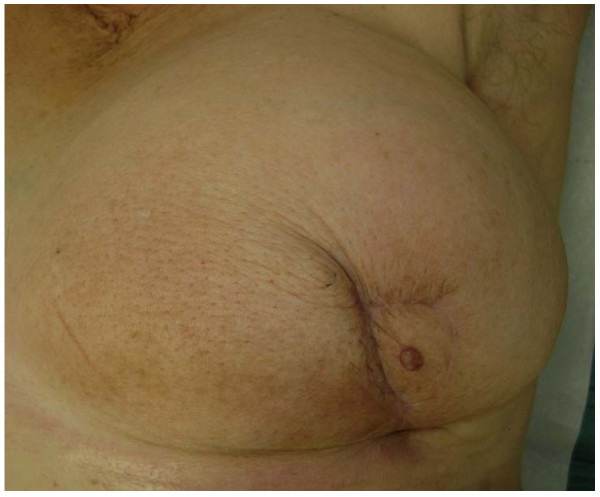
**Results at 6 months after surgery**.

After six months from the operation, the breast results smaller than the contralateral, but still presents an acceptable shape with a fair projection of the reconstructed nipple, though all the treatments the patient underwent so far (Fig. 3).

The final histological result showed a fibrotic mastopathy without atypias.

## Discussion

Nipple dichotomy (or intra-areolar polythelia) is an extremely rare malformation in which one or more supernumerary nipples are located within the same areola. It is congenital and probably due to an intrauterine division of the developing breast. It must be differentiated from a much more common condition called polythelia, in which the supernumerary nipples typically occur on the "milk line", in the vertical axis of the ventral surface of the thorax and abdomen, from the axillae to the groin, regarded as atavistic remnants of the mammary ridges during embryogenesis [1]. The milk line, in fact, becomes evident at 6 weeks gestation and regresses by the 10^th ^week of intrauterine development. No endocrine dysfunction is associated to this malformation and the embryological mechanisms that underlie its development remain unknown. Only in a few series, an increased frequency of urinary tract malformation has been documented in patients with supernumerary nipples [2,3].

In the sporadic cases described in the Literature (only 11 cases reported), nipple dichotomy is usually bilateral, often associated with hypoplastic breasts [4]. Familial cases are also frequent [5,6]. In the present case, no familial or personal history of other congenital malformations have been reported by the patient.

The nipple-sharing technique in the reconstruction of the nipple after mastectomy or central quadrantectomy is simple and quick, can be performed under local anaesthesia, such in this case, and usually provides good symmetry with minimal donor site morbidity [7,8].

## Conclusion

In the present patient, affected by a rare and benign abnormality known as nipple dichotomy, the nipple sharing technique allowed in the same time the correction of the congenital defect and provided the surgeon with a supernumerary nipple to be used in the immediate reconstruction.

No other study in the Literature reports the use of a supernumerary nipple in the reconstruction of a contralateral breast after a conservative approach for cancer; the final result, provided the patient had a double surgery and radiation therapy's sequelae, is to be considered acceptable in terms of breast volume and reconstructed nipple projection.

## Consent

Written informed consent was obtained from the patient for publication of this case report and accompanying images. A copy of the written consent is available for review by the Editor-in-Chief of this journal.

## Competing interests

The authors declare that they have no competing interests.

## Authors' contributions

All of the named authors were involved in the preparation of this manuscript. All authors read and approved the final manuscript.
